# CSF and Serum Biomarkers Focusing on Cerebral Vasospasm and Ischemia after Subarachnoid Hemorrhage

**DOI:** 10.1155/2013/560305

**Published:** 2013-02-19

**Authors:** Carla S. Jung, Bettina Lange, Michael Zimmermann, Volker Seifert

**Affiliations:** ^1^Department of Neurosurgery, Johann-Wolfgang Goethe University, 60528 Frankfurt, Germany; ^2^Department of Neurosurgery, Ruprecht-Karls University of Heidelberg, 65120 Heidelberg, Germany

## Abstract

Delayed cerebral vasospasm (CVS) and delayed cerebral ischemia (DCI) remain severe complications after subarachnoid hemorrhage (SAH). Although focal changes in cerebral metabolism indicating ischemia are detectable by microdialysis, routinely used biomarkers are missing. We therefore sought to evaluate a panel of possible global markers in serum and cerebrospinal fluid (CSF) of patients after SAH. CSF and serum of SAH patients were analyzed retrospectively. In CSF, levels of inhibitory, excitatory, and structural amino acids were detected by high-performance liquid chromatography (HPLC). In serum, neuron-specific enolase (NSE) and S100B level were measured and examined in conjunction with CVS and DCI. CVS was detected by arteriography, and ischemic lesions were assessed by computed tomography (CT) scans. All CSF amino acids were altered after SAH. CSF glutamate, glutamine, glycine, and histidine were significantly correlated with arteriographic CVS. CSF glutamate and serum S100B were significantly correlated with ischemic events after SAH; however, NSE did not correlate neither with ischemia nor with vasospasm. Glutamate, glutamine, glycine, and histidine might be used in CSF as markers for CVS. Glutamate also indicates ischemia. Serum S100B, but not NSE, is a suitable marker for ischemia. These results need to be validated in larger prospective cohorts.

## 1. Introduction

Besides acute brain injury [1], one-third of patients suffering from subarachnoid hemorrhage (SAH) develop secondary brain injury [2]. This secondary brain injury leading to the majority of morbidity and mortality after SAH seems to be due to delayed cerebral vasospasm (CVS), which results in delayed cerebral ischemia (DCI) [3]. There are a number of other causes of cerebral ischemia other than CVS after SAH [4], which may manifest clinically as delayed ischemic neurological deficits (DINDs).

CVS has been associated with DIND and DCI and was described for a long time as the underlying pathophysiology [5–8]. However, recent studies showed that ameliorating CVS is only partially effective in preventing DCI [9]. This might be explained by multifactorial mechanisms underlying DCI and the development of secondary brain injury. It further implies SAH and biomarker research aiming at a more comprehensive detection of secondary events after SAH; that one should not focus solely on CVS, but rather on evaluating CVS and DCI.

Although extensive research has been conducted over the last decades on monitoring tissue biochemistry in the injured brain and some studies have identified predictors of CVS following SAH (for review, see Lad et al., 2012 and Table 1) [10], no biomarkers predictive of CVS, DCI, or outcome have been incorporated into routine clinical work. Cerebral microdialysis has been demonstrated to be a useful method detecting biochemical changes associated with brain ischemia after acute brain injury [11]. Especially, the excitatory amino acid glutamate (Glu) has been predictive of ischemia [12]. However, microdialysis remains a focal indicator for intracerebral events, and its distribution and use among ICUs worldwide are limited [11]. Therefore, we sought to evaluate a possible panel of biomarkers in CSF and serum, including excitatory, inhibitory, and structural amino acids as well as neuron-specific enolase and S100B, which might facilitate to detect CVS and/or DCI after SAH and might help to explain pathophysiological changes related to secondary brain injury after SAH.

## 2. Material and Methods

Stored serum and cerebrospinal fluid (CSF) samples of patients suffering from aneurysmal SAH (*n* = 18) and of controls (*n* = 5) with hydrocephalus after intracerebral hemorrhage, but without aneurysmal SAH, tumor, or trauma, were retrospectively analyzed. All SAH patients (*n* = 18) were Fisher grade III or IV [13] and had suffered from acute hydrocephalus after SAH which was treated by early placement of an external ventricular drainage (EVD) before or during aneurysm treatment. In control patients, a single CSF sample was collected during placement of EVD. Samples were immediately centrifuged, and supernatants were stored at –70°C until further assessment. Samples were collected between days 0 and 12 after SAH, depending on how long the EVD remained in situ. For 3 patients no CSF was accessible past day 9 after SAH. As stored CSF samples were also not accessible for every day, short time periods of 3 days each were defined: days 0–3, 4–6, 7–9, and 10–12 after SAH. Of every patient one sample was taken within each of these time periods for analysis. Additional selection criteria for samples drawn within each of the 3-day time periods were, that CSF collected at the day of arteriography and at the day CT scans were performed had to be available and were included for analysis. Serum samples were collected daily during intensive care stay and analysed from the first sample taken after admission until day 12 after SAH.

Sample collection and retrospective analysis were approved by the ethics committee of the University of Frankfurt/Main. 

### 2.1. Biomarker Detection

In serum neuron-specific enolase (NSE) and S100B protein were determined using LIAISON Sangtec 100 assay and LIAISON NSE assay (Byk-Sangtec Diagnostica, Germany). In CSF high performance liquid chromatography (HPLC) was performed to detect the levels of free amino acids including the excitatory amino acids: aspartate (Asp) and glutamate (Glu), as well as the inhibitory amino acids: glycine (Gly) and *γ*-aminobutyric acid (GABA). Furthermore, the structural, nontransmitter AA glutamine (Gln), histidine (His), and serine (Ser) were detected. Chromatography conditions and quantification were previously described [14].

### 2.2. Clinical Assessment and Detection of Delayed Cerebral Vasospasm (CVS) and Cerebral Ischemia (DCI)

Patients suffering from aneurysmal SAH were examined at admission using the Hunt and Hess classification [15] and the World Federation of Neurological Surgeons SAH scale (WFNS scale) [16] as well as at discharge using the Glasgow Outcome Scale (GOS) [17]. 

All patients underwent either early clipping (*n* = 13) or coiling (*n* = 5) of the detected ruptured aneurysm, within 72 hours after the initial bleed, followed by hypertensive hypervolemic hemodilution therapy to prevent vasospasm-induced brain ischemia.

Delayed cerebral vasospasm (CVS) was detected arteriographically: an early baseline cerebral arteriography, performed between days 0 to 2 after SAH, was compared with a subsequently performed arteriography 7 ± 1 days after SAH. The time point of the second arteriography depended on the individual clinical course and was influenced by clinical symptoms and transcranial Doppler sonography (TCD) signs for cerebral vasospasm (increase in flow velocity >30 cm/sec compared to previous days or an overall increase >200 cm/sec). Arteriographic CVS was quantified relative to each patient's baseline arteriogram and was measured by two blinded examiners as described previously. CVS was graded as none, mild, moderate, or severe arteriographic cerebral vasospasm [18]. 

Delayed cerebral ischemic events (DCIs) were assessed by follow-up computed tomography (CT) scans and determined as hypointensive changes reflecting partial or total involvement of the territory of a cerebral artery on CT scans [19]. To differentiate between treatment-induced ischemic events and SAH-induced delayed cerebral ischemia (DCI) a CT scan was performed within 24 hours after clipping or coiling. DCI included all ischemic lesions detected in subsequent follow-up CT scans, more than 24 hours after treatment. Cerebral ischemia was graded 0 if no hypointensive changes were detected. A small perforator infarction was graded as I and a territorial infarction as grade II.

### 2.3. Statistical Analysis

Data are presented as mean value ± standard deviation (SD). Statistical analysis of the data was performed using two-tailed Student's *t*-test and analysis of variance (ANOVA) followed by Tukey's test for *post hoc* comparisons of mean values. Pearson's correlation coefficient was used to assess correlations. Statistical significance was defined as *P* < 0.05.

## 3. Results

Of 18 retrospectively analyzed SAH patients, thirteen developed arteriographic CVS. 6 patients showed cerebral ischemic events which were related to treatment and visible in the early CT scans 24 hours after aneurysm treatment. All treatment-related infarctions were perforator infarctions and were classified as grade I. One patient with a treatment-related perforator infarction developed CVS. Follow-up CT scans of this patient revealed a territorial infarction in the distribution of the formerly detected CVS. Altogether, five patients developed DCI on computed tomography several days after clipping or coiling. These infarctions were all big territorial infarctions (grade II). None of the patients showed small, grade I delayed cerebral ischemia in follow-up CT scans. All patients who developed DCI suffered also from moderate or severe arteriographic vasospasm. Furthermore, patients without signs of arteriographic vasospasm showed no delayed ischemic events on follow-up CT scans (Table 2). Clinical examination at admission (WFNS grade as well as Hunt and Hess grade) was not correlated with outcome measures (GOS) at discharge. 

### 3.1. Biomarkers

**Figure 1:**
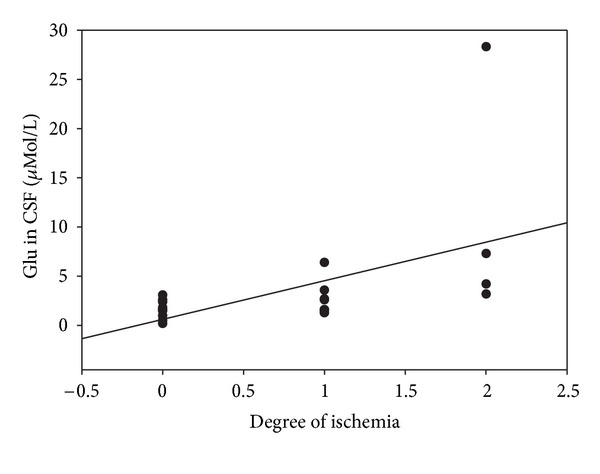
The graph depicts CSF Glu in association with the degree of ischemia.

CSF glutamine (Gln), glycine (Gly), serine (Ser), and histidine (His) concentrations significantly increased after SAH. CSF *γ*-aminobutyric acid (GABA) significantly decreased compared to control values (0.22 ± 0.13 *μ*mol/L) after an initial increase (20.6 ± 36.4 *μ*mol/L, *n* = 18) on days 0–3 after SAH. Glutamate (Glu) showed in all SAH patients a trend to increase, which did not reach statistical significance. Furthermore, aspartate (Asp) remained unchanged after SAH. However, Glu (CC: 0.48; *P* = 0.03), Gln (CC: 0.47; *P* = 0.04), Gly (CC: 0.53; *P* = 0.02), and His (CC: 0.66; *P* = 0.001) were correlated with the occurrence of arteriographic CVS at the day arteriography was performed. In addition Glu was correlated with the size of ischemia (CC: 0.51; *P* = 0.02) (Figure 1) on the day CT scans are performed. However, no difference could be observed between treatment-related ischemia or SAH-related DCI. Although Ser significantly increased after SAH, it showed in addition to Asp no correlation with CVS or ischemia.

**Figure 2:**
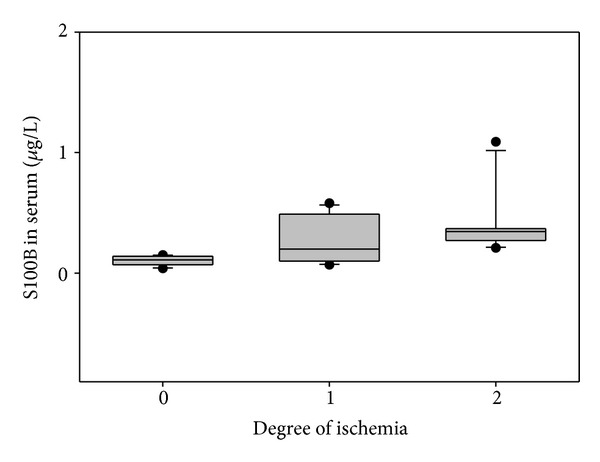
The graph depicts S100B in serum in association with the degree of ischemia.

In serum, no significant changes could be detected for S100B during the time course after SAH, and no correlation was detectable with the development of CVS on the day arteriography was performed (CC = 0.51; *P* = 0.052). However, S100B serum levels were associated with ischemic events detected in follow-up CT scans, irrespective of whether the ischemic lesion was treatment-related or supposedly SAH-related DCI. Serum S100B concentrations were further correlated with the size of ischemic lesions (CC = 0.54; *P* = 0.03) (Figure 2). However, NSE in serum was neither correlated with CVS (*P* = 0.3) nor with ischemia (*P* = 0.7). 

No association could be detected between clinical parameter (WFNS as well as Hunt and Hess grades) and the different CSF amino acid (AA) levels at admission. Furthermore, no correlation was detectable between CSF AA levels and GOS outcome parameter at discharge. 

## 4. Discussion

Secondary brain injury exacerbating morbidity and mortality after SAH seems to be due to CVS and DCI. Delayed ischemic neurological deficits (DINDs) seem to result from tissue ischemia. In addition, DIND and DCI have been associated with vascular territories in which CVS has been documented arteriographically, suggesting a causal relationship [5–8]. In accordance with these former studies, 5 patients who derived from the SAH group with arteriographic vasospasm developed DCI, detected as delayed ischemic lesions on follow-up CT scans. Recent reports, however, on CVS after SAH cast doubt on the assumption that DCI is caused only by CVS [25, 26]. Vergouwen et al. showed that cerebral infarction after SAH had a direct effect on outcome independent of arteriographic CVS and suggested that coexisting factors might be involved in the pathogenesis of DIND and DCI [27]. For example, delayed spreading ischemia was suggested as an additional possible source of DIND [28–30]. However, data of this study do not suffice to give any hint concerning this hypothesis. In particular, we did not use MRI to detect ischemic lesions. Furthermore, Jordan and Nyquist proposed that CVS could be an epiphenomena or a contributing factor to parenchymal destruction [31]. In accordance with these observations detected associations between CSF amino acids and CVS, as seen for Gln, Gly, and His, are often not accompanied by an association with DCI. Although we agree that an association between CVS and DCI does not prove a causal relationship, it seems conspicuous that only patients with arteriographic vasospasm developed DCI detected as delayed ischemic lesions on follow-up CT scan in this study. In this perspective, monitoring brain injury and clinical course after SAH demands biomarkers to detect delayed cerebral vasospasm and delayed cerebral ischemia/infarctions. To identify impending secondary injury and to explain neuropathological changes, markers reflecting global processes within the brain, as expected from CSF and serum markers, could be advantageous.

### 4.1. CSF Marker

After traumatic brain injury the release of the excitatory amino acid glutamate (Glu) and aspartate (Asp), measured in interstitial fluid of the brain and in CSF, was strongly correlated with increased ICP, secondary brain injury, and poor outcome [32–34]. Asp and Glu have been reported as markers of cellular degradation [35], and Glu has been discussed as a predictive biomarker for secondary brain injury and has been demonstrated to be a useful parameter in microdialysis for detection of brain ischemia after SAH [11, 12]. Consistent with this observation Glu CSF concentrations were correlated with CVS and DCI in this study. Excitotoxicity has been suggested as a mechanism of ischemic secondary brain injury, mediated by excessive calcium influx via glutamate-mediated ion channels [33]. Glu further participates in multiple biochemical pathways. It plays a role in neuron-glia communications: the released Glu is taken up into the glia and is converted to glutamine (Gln) which is transported back to the presynaptic neuron and then reconverted to Glu. Glu and Gln CSF concentrations detected were comparable to those described previously [36]. Furthermore, GABA derives from Glu and vice versa. Therefore, alterations in glutamate metabolism might take effect on GABA metabolism [36]. Hutchinson et al. described increased GABA levels, measured by microdialysis, in SAH patients who suffered from DCI, while GABA levels under basal conditions were low. In addition a correlation between GABA and Glu was observed [37]. In contrast CSF Glu increased and CSF GABA decreased after SAH in this patient collective, and no association could be observed. This difference, between microdialysis and CSF examination results, might be due to rapid clearance and limited diffusion of GABA from its neuronal and synaptic origin [38, 39].

Glycine (Gly) also belongs to the inhibitory amino acids and represents the major amino acid found in collagens and thus in cell membranes. Next to its function as precursor of a variety of metabolic products serine (Ser) is also found in high concentration in cell membranes [40]. Histidine is an essential amino acid and the precursor of histamine. Furthermore it is involved in synthesis of hemoglobin. Because of its free radical scavenging characteristics it was reported to attenuate CVS in a rabbit model of SAH [41]. The release and increase of these structural amino acids might be an indicator for progressive cell membrane degradation. Under experimental conditions excitatory amino acids release has normalized rapidly after global ischemia with reperfusion [42]. Ischemia-induced release of neuroactive amino acids has been suggested to result from energy substrate depletion which is related to reduction in regional blood flow [43], leading to a Ca^2+^-dependent efflux of neurotransmitters [44] and to inhibition of the neurotransmitter uptake system [45]. Therefore, the more blood flow is reduced, the more efflux of amino acids is expected. Thus, excitatory and inhibitory amino acid detection should increase. 

### 4.2. Serum Marker

S100B and NSE in serum have been discussed as prognostic marker after SAH [21–24] (Table 1). Oertel et al. tried to predict CVS and outcome within the first 3 days after SAH by measuring S100B in serum. Although they did not succeed to differentiate between favorable and unfavorable outcomes, they found significantly higher S100B levels in serum in patients who did not develop CVS as well as in those who died [21]. Moritz et al. showed that serum S100B but not serum NSE allows for determination of good and bad outcomes after SAH [24]. Furthermore, serum S100B allowed the detection of cerebral infarction but not of CVS [24]. Although the low number of patients with grade II ischemia as well as the variance in samples led only to a weak correlation between S100B and degree of ischemia in this study, we could confirm S100B in serum as an indicator for cerebral ischemia. S100B concentrations above 0.15 *μ*g/L were associated with the occurrence and size of ischemic lesions in follow-up CT scans. Similar to Moritz et al., S100B was not associated with arteriographic CVS, and serum NSE did not correlate neither with ischemia nor with vasospasm. Therefore, NSE seems not to be useful as a biomarker for monitoring SAH patients. Herrmann et al. measured S100B after acute stroke using the same assay. They reported that patients who suffered from stroke which was completely reversible within a few days had no increased serum S100B levels. These findings are comparable with those of patients of this study who develop CVS but showed no DCI in follow-up CT scans suggesting a possible pathophysiological point of no return in DCI development from CVS. The fact that S100B is correlated only with ischemic events and not with arteriographic assessed CVS points to possible different degrees of tissue degradation among the wide range of CVS going from reversible mild narrowing to severe constriction leading to ischemia needed to detect S100B in serum. In manifest stroke serum S100B levels described a decelerated increase compared with GFAP [20]. The different expression patterns have been explained by different release patterns under pathological conditions: necrotic cell death leading to leakage from cytosol, breakdown of membrane integrity in the penumbra of infarcts due to cytotoxic, and vasogenic edema as well as brain repair mechanisms [20].

### 4.3. Limitations of the Study

In this study, selection criteria as, for example, Fisher Grade III and IV and acute hydrocephalus, might lead to study bias. Furthermore, no MRI data could be used to assess ischemia because of the retrospective nature of this study. In addition, the cohort is relatively small and contains only a small amount of patients with DCI, matching the usual distribution of DCI after SAH. In addition, outcome of the patients was assessed at discharge. Therefore the results are limited to short-term and not to long-term outcome. Furthermore, WFNS grade as well as Hunt and Hess grades was not correlated with short-term outcome parameter GOS at discharge. This may be due to the small number of patients in each WFNS/Hunt and Hess subgroup or the limitation of this study to only “poor grade” patients. 

## 5. Conclusions

After SAH glutamate, glutamine, glycine, and histidine might in addition to microdialysis be used in CSF as markers for arteriographic CVS. Glutamate also indicated ischemia. Serum S100B, but not NSE, was associated with delayed cerebral ischemia, but was not correlated with arteriographic CVS. These results need to be validated in a larger prospective cohort.

## Figures and Tables

**Table 1:** Summary of the literature focusing on S100B and NSE as biomarkers after SAH predictive of CVS and/or DCI.

Author	Year	Serum biomarker	Sample collection	CVS assessment	CVS	DCI assessment	DCI	Bad outcome
Herrmann et al. [20]	2000	S100B	First 4 days after ischemic stroke	NE	NE	CT	++	++
		No NSE detection			NE	for ischemia detection	(volume of lesion)	

Oertel et al. [21]	2006	S100B	First 3 days after SAH	TCD	↑	NE	NE	+↑
		NSE				NE	NE	↓

Weiss et al. [22]	2006	S100B	First 8 days after SAH	TCD + arteriography	−	NE	NE	++
		No NSE detection			(only CVS + S100B < 0.4 *μ*g/L: no death)			

Sanchez-Pe$\tilde{\text{n}}$a et al. [23]	2008	S100B	First 15 days after SAH	TCD + arteriography	↑ in “ischemic vasospasm” patients			++ (↑)
		No NSE detection						(only mean 15 day S100B value)

Moritz et al. [24]	2010	S100B	Daily during ICU stay	TCD	−	CT	++	++
		NSE			−	CT	+	+ (only NSE peak value)

NE: not evaluated; ↑: increase; ↓: decrease; “−”: no correlation; “+”: positive correlation; “++”: prognostic factor; TCD: transcranial doppler sonography;

**Table 2:** Summary of patient characteristics.

Patient	Gender	CVS	DCI	Pop ischemia
1	M	+	+	−
2	M	+	+	+
3	M	+	+	−
4	M	+	−	−
5	F	+	+	−
6	F	+	−	−
7	F	+	+	−
8	M	+	−	−
9	M	+	−	−
10	M	+	−	−
11	F	+	−	−
12	F	+	−	+
13	F	+	−	+
14	M	−	−	+
15	F	−	−	+
16	F	−	−	−
17	F	−	−	−
18	F	−	−	−

M: male; F: female; CVS: cerebral vasospasm; DIND: delayed ischemic neurological deficit detected in alert patients; DCI: delayed cerebral ischemia; pop ischemia: postoperative/treatment-related ischemia; “+”: with; “−”: without the characteristic measure.
